# Flow-induced HDAC1 phosphorylation and nuclear export in angiogenic sprouting

**DOI:** 10.1038/srep34046

**Published:** 2016-09-27

**Authors:** Despina Bazou, Mei Rosa Ng, Jonathan W. Song, Shan Min Chin, Nir Maimon, Lance L. Munn

**Affiliations:** 1 Edwin L. Steele Laboratories, Department of Radiation Oncology, Massachusetts General Hospital and Harvard Medical School, 100 Blossom Street, Boston, Massachusetts 02114, USA; 2 Department of Mechanical and Aerospace Engineering, Ohio State University, E406 201 W. 19th Avenue, Columbus, OH 43210, USA

## Abstract

Angiogenesis requires the coordinated growth and migration of endothelial cells (ECs), with each EC residing in the vessel wall integrating local signals to determine whether to remain quiescent or undergo morphogenesis. These signals include vascular endothelial growth factor (VEGF) and flow-induced mechanical stimuli such as interstitial flow, which are both elevated in the tumor microenvironment. However, it is not clear how VEGF signaling and mechanobiological activation due to interstitial flow cooperate during angiogenesis. Here, we show that endothelial morphogenesis is histone deacetylase-1- (HDAC1) dependent and that interstitial flow increases the phosphorylation of HDAC1, its activity, and its export from the nucleus. Furthermore, we show that HDAC1 inhibition decreases endothelial morphogenesis and matrix metalloproteinase-14 (MMP14) expression. Our results suggest that HDAC1 modulates angiogenesis in response to flow, providing a new target for modulating vascularization in the clinic.

The formation of blood vessels through the process of angiogenesis is critical for normal vascular development but is also implicated in numerous pathologies including tumor growth and metastasis^1^,^2^. Angiogenesis requires the coordinated growth and migration of endothelial cells (ECs), which remain connected with the vessel wall. The ECs have to integrate multiple local signals to determine whether to remain quiescent, participate in dilation or contraction^1^, or undergo morphogenesis to form an angiogenic sprout^3^,^4^,^5^,^6^ or intussusceptive involution^7^. Biochemical stimuli include various growth factors (FGF, TNF-α, IGF), with vascular endothelial growth factor (VEGF) playing a key role by regulating the survival, proliferation and migration of ECs^8^.

Besides biochemical stimuli, flowing blood and plasma can activate mechano-sensors to modulate EC morphology and function through independent or complementary signaling pathways^9^,^10^,^11^. ECs in blood vessels can be influenced by mechanical forces tangential to the endothelial surface due to blood flow^5^,^12^ and across the vessel wall due to interstitial plasma flow^13^,^14^. Using a microfluidic model of angiogenic sprouting, we have previously shown that fluid shear stress inhibits vessel morphogenesis via the nitric oxide (NO) pathway, whereas interstitial flow increases the rate of morphogenesis and anastomosis^15^,^16^,^17^.

Although there have been numerous studies on the mechanisms involved in modulating EC responses to different flow patterns and shear stresses, there is a lack of information on the role of epigenetic factors in modulating EC mechanotransduction^18^. Epigenetic modifications can modulate gene expression without changing the DNA sequence, and thus provide rapid and reversible regulation of the repertoire of expressed genes^19^. One important epigenetic modification involves histone acetylation/deacetylation, which can directly control gene expression^20^. Histone acetyltransferases stimulate transcription though acetylation of histones, resulting in relaxation of nucleosomes; conversely, histone deacetylases (HDACs) promote histone deacetylation and repress transcription by condensing the chromatin^21^,^22^. HDACs are classified into four main groups: Class I (HDAC-1/2/3 and HDAC8), class II (HDAC-4/5/6/7 and HDAC-9/10), class III sirtuins (SIRT; *i.e*. SIRT1-7) and class IV (HDAC-11)^23^,^24^. There is increasing evidence that members of the HDAC family play important roles in EC differentiation, as well as in atherogenesis in regions of disturbed flow^25^,^26^,^27^,^28^,^29^,^30^. However, little is known about their role in sprouting angiogenesis in response to transwall flow stimuli that are prevalent in hyperpermeable vessels during inflammation or tumor growth.

In this study, we provide new insight into the role of post-translational modifications induced by mechanical forces in vascular endothelial cell morphogenesis. We focus on the role of interstitial flow, as it is a poorly understood modulator of vessel morphogenesis. We show that angiogenic morphogenesis is HDAC1-dependent and that interstitial flow increases the phosphorylation of HDAC1, its activity, and its export from the nucleus. Furthermore, we show that MMP14, a protease involved in angiogenic sprouting, is regulated by HDAC1, and that inhibition of HDAC1 nuclear export downregulates MMP14 protein expression. Our results suggest the existence of flow-dependent and -independent pathways involving VEGF and HDAC1 that contribute to vascular morphogenesis.

## Results

### Angiogenic sprouting and invasion *in vitro*

**Figure 1:**
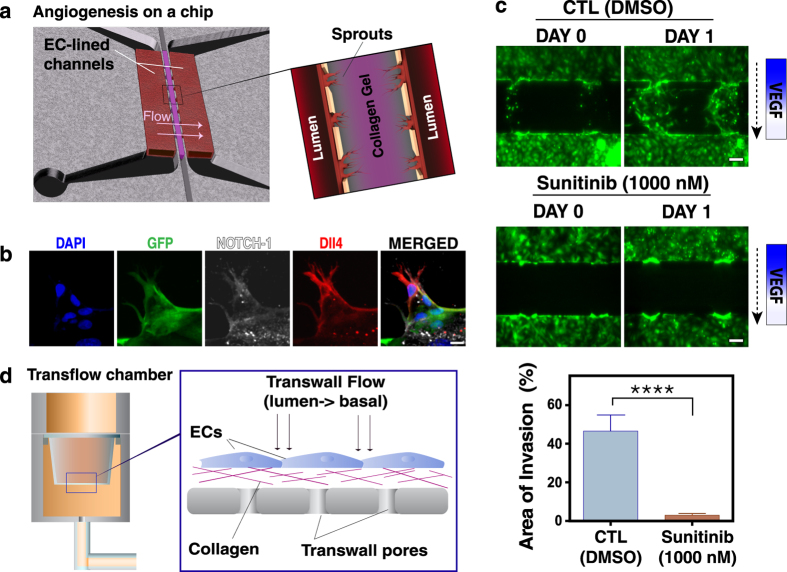
Angiogenic sprouting in a microfluidic device. (**a**) Schematic of the PDMS microfluidic device. HUVECs are seeded into two channels (red) separated by the polymerized collagen gel (purple). Transendothelial interstitial flow (arrow) is applied across the collagen gel. Each HUVEC channel has independent input and outlet ports allowing strict flow control in both channels. Close - up view of the boxed area shows three apertures that allow invasion and sprouting of the lumenized vessel segments (red) into the central collagen gel (purple). (**b**) Immunofluorescence staining for Notch-1 (white) and Dll4 (red) expression in GFP transduced HUVECs sprouting in 3D collagen. Nuclei were stained with DAPI (Scale bar, 25 μm). (**c**) Angiogenic sprouting and invasion into the 3-D collagen matrix under interstitial flow (dashed arrow indicates flow direction) and a VEGF gradient is significantly inhibited in response to 1000 nM of sunitinib. Control devices treated with DMSO proceeded with extensive sprouting (Scale bar, 100 μm). Data represent mean ± SEM, ****p < 0.0001. (**d**) Schematic of the transwell macroscale device. Collagen and fibronectin were loaded into a 6-well transwell chamber and HUVECs (10^6^/ml) in EGM media were allowed to spread and form a confluent monolayer for 24 h prior to flow initiation. Interstitial flow (0.5 μm/sec) across the HUVEC monolayer was driven using a programmable syringe pump (Harvard Apparatus). Boxed area shows a close-up schematic of interstitial flow imposed across the 6-well transwell. Flow is driven through the EC junctions, collagen gel substrate and the transwell ports analogous to an apical/lumen - basal flow.

To determine how interstitial flow modulates angiogenic morphogenesis, we used our previously described microfluidic device that provides control of fluid convection through bio-mimetic vessels^17^. In this device, human umbilical vein endothelial cells (HUVECs) form parallel vessels separated by a 3-dimensional (3D) collagen I/fibronectin matrix (Fig. 1a). HUVECs stimulated with 50 ng/ml VEGF migrate into the 3D matrix to create new lumenized vessels that eventually connect the two pre-defined vessels (Fig. 1a, schematic), thus mimicking angiogenic sprouting and anastomosis. In this process, ECs extend filopodial extensions analogous to tip cell sprouts seen *in vivo*^31^,^32^. The extending sprouts display the ‘typical’ molecular signature of sprouting cells: increased Delta-like ligand-4 (Dll4) staining in tip cells, while the expression of Dll4 is reduced in the trailing stalk cells (Fig. 1b). In contrast, Notch-1 is expressed diffusely in stalk cells (Fig. 1b), consistent with the mechanism of tip-stalk cell communication previously reported^4^. In addition, these endothelial sprouts respond appropriately to anti-angiogenic treatment. Addition of sunitinib, which blocks VEGFR2 phosphorylation at Tyr951, inhibits invasion into the collagen matrix (Fig. 1c).

To identify potential phosphorylation events associated with the early response to flow stimuli, we performed a protein phosphorylation profiling and screening array on ECs exposed to flow for only 1 h. For these studies, we used a macroscale device (Fig. 1d)^15^,^33^,^34^, which allowed stimulation of larger numbers of endothelial cells and collection of sufficient protein samples. The most dramatic phosphorylation event detected in this array was in HDAC1, which increased 17-fold, prompting us to further investigate the role of HDAC1 in flow-induced angiogenic morphogenesis.

### HDAC1 is involved in flow-induced morphogenesis

**Figure 2:**
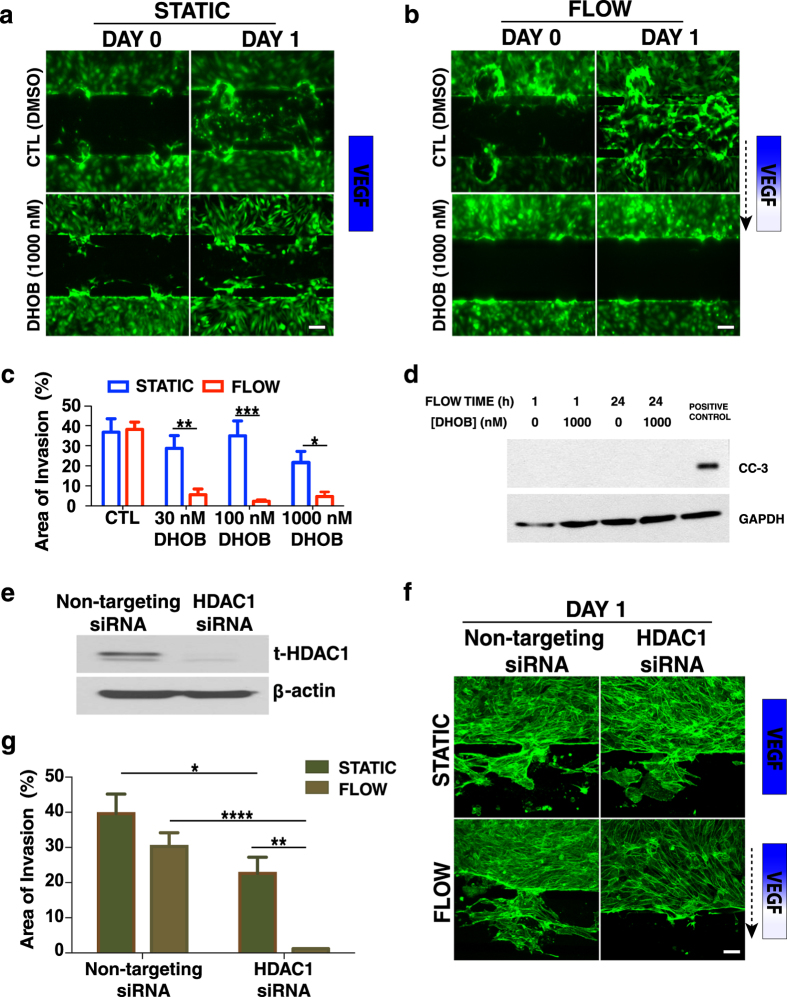
HDAC1 enables angiogenic invasion under flow. Blocking HDAC1 with the selective HDAC1 inhibitor DHOB (1000 nM) for 24 h (**a**) under static conditions (without a VEGF gradient) has no effect on angiogenic invasion, while (**b**) in the presence of interstitial flow and a VEGF gradient sprouting is significantly reduced. The dashed arrow indicates the flow direction. Control devices (treated with DMSO) showed significant invasion under static and flow conditions. (**c**) Quantification of invasion (displayed as the % of spout area) after one day of continuous DHOB treatment under static and flow conditions. Data represent mean ± SEM, *p < 0.05, **p < 0.01, ***p < 0.001. (**d**) DHOB at 1000 nM did not promote EC apoptosis after 1 and 24 h of continuous perfusion as determined by western blot analysis for cleaved caspase 3. (**e**) Knockdown of HDAC1 was achieved by siRNA transfection. (**f**) Silencing HDAC1 with siRNA under static (no VEGF gradient) conditions (top row) has a small effect on angiogenic sprouting, while in the presence of interstitial flow and a VEGF gradient (bottom row) sprouting is significantly reduced. Exogenous VEGF (50 ng/ml) was also included in the media. The dashed arrow indicates the flow direction. In contrast, control devices with cells transfected with non-targeting siRNA proceeded with significant invasion under static and flow conditions. (**g**) Quantification of the % of invasion area after one day of under static and flow conditions for HDAC1 and non-targeting siRNA treated endothelial cells. Data represent mean ± SEM, *p < 0.05, **p < 0.01, ****p < 0.0001.

We next investigated whether HDAC1 plays a role in endothelial cell morphogenesis in response to interstitial flow in our microfluidic device. ECs were continuously treated with an inhibitor of HDAC1, 4-(dimethylamino)-N- [6-(hydroxyamino)-6-oxohexyl]-benzamide (DHOB), for 24 h in the presence of interstitial flow or under static conditions. Exogenous VEGF (50 ng/ml) was also included in the media. ECs in control (CTL DMSO) devices invaded under static and flow conditions (Fig. 2a–c). DHOB inhibited morphogenic invasion under flow conditions, but not in static devices. These results suggest that endothelial morphogenesis is HDAC1-dependent in the presence of interstitial flow but HDAC1-independent under static conditions. In these experiments, DHOB did not induce cell apoptosis: DHOB at the highest concentration employed (1000 nM) did not induce cleaved caspase-3 (CC-3) over 24 h of continuous perfusion (Fig. 2d).

To further validate our results with the DHOB inhibitor, we selectively knocked down HDAC1 using siRNA (Fig. 2e). As in the pharmacological blocking experiments, invasion was significantly reduced under flow when HDAC1 was genetically suppressed (Fig. 2f,g). Under static conditions there was a smaller, but significant, inhibition of invasion by the HDAC1 knockdown cells, indicating that HDAC1 may also be involved in static sprouting or that there was some residual convective flow in the microdevices, even without imposed flow. Together, our results strongly suggest that HDAC1 mediates EC morphogenesis in response to mechanical flow stimuli.

### Interstitial flow stimulates HDAC1 phosphorylation and increased HDAC1 activity

**Figure 3:**
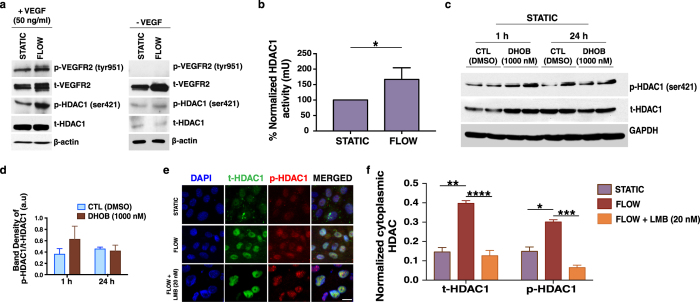
Interstitial flow-induced HDAC1 phosphorylation and nuclear export in endothelial cells. (**a**) Representative western blot showing that in the presence of exogenous VEGF (50 ng/ml), interstitial flow leads to increased HDAC1 phosphorylation at Ser421. In the absence of exogenous VEGF and consequently of VEGFR2 phosphorylation, no significant difference could be detected in HDAC1 phosphorylation under flow relative to static. The blot was cropped, and the full-length blot is presented in Supplementary Fig. S1. (**b**) HDAC1 activity (mU) assay showing a 67% increase in HDAC1 activity under flow relative to static. Data are normalized and shown as % increase in HDAC1 activity under flow conditions relative to static (set as 100%). (**c**) Western blot analysis (duplicates are shown) and (**d**) band density quantification showing that in the presence of DHOB (1000 nM) for 1 and 24 h, phosphorylation of HDAC1 at Ser421 is not inhibited. The blot was cropped and the full-length blot is presented in Supplementary Fig. S1. (**e**) Immunofluorescence staining showing increased p-HDAC1 outside the nuclear (DAPI positive) area under flow (*middle row*) in relation to static (*top row*). LMB (20 nM) treatment blocked the increase (*bottom row*) (Scale bar, 20 μm). (**f**) Quantification of the area fraction of cytoplasmic t- and p-HDAC1 under static, flow and flow + LMB (20 nM) conditions. Data represent mean ± SEM, *p < 0.05, **p < 0.01, ***p < 0.001, ****p < 0.0001.

To examine the mechanism by which HDAC1 modulates angiogenic morphogenesis, we subjected ECs to interstitial flow in the macroscale transwell device. HDAC1 is known to be phosphorylated at Ser421, and this phosphorylation increases its deacetylation activity^35^. In the presence of VEGF, interstitial flow increased HDAC1 phosphorylation at Ser421 relative to static cultures, suggesting that the phosphorylation of HDAC1 is mechanically regulated (Fig. 3a). In contrast, in the absence of VEGF, and thus in the absence of phosphorylation of VEGFR2, there was no difference in HDAC1 phosphorylation under flow relative to static conditions (Fig. 3a). HDAC1 phosphorylation was accompanied by a 67% increase in its activity (Fig. 3b). These data indicate that phosphorylation, and thus increased activity of HDAC1, plays a role in VEGF- and interstitial flow-induced endothelial morphogenesis.

### HDAC1 phosphorylation at Ser421 is not affected by DHOB treatment

We next investigated whether DHOB treatment affects HDAC1 phosphorylation. ECs were cultured in the absence of flow, treated with DHOB for 1 and 24 h, and then protein was collected for Western blot analysis. DHOB did not affect HDAC1 phosphorylation (Fig. 3c,d), indicating that it interferes with some other aspect of HDAC1 function. The increased activity of HDAC1 could potentially be due to a change in its association with its co-repressor complex. In fact, DHOB belongs to the benzamide class of HDAC inhibitors most likely acting as a zinc-chelating moiety to alter the active site of HDAC1^20^.

### Interstitial flow alters HDAC1 sub-cellular localization

We next investigated whether the subcellular localization of HDAC1 changes during flow-induced endothelial cell morphogenesis. Although HDAC1 is classified as a nuclear protein, its localization in the cytoplasm has been reported to have functional significance^36^,^37^.

**Figure 4:**
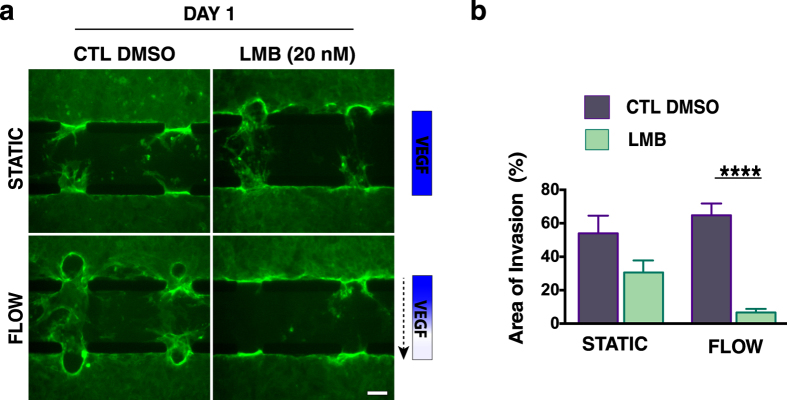
Inhibition of p-HDAC1 nuclear export inhibits angiogenic invasion. (**a**) Angiogenic invasion into the 3-D collagen matrix under static conditions (no VEGF gradient) (top row) is not inhibited by a 24 h LMB (20 nM) treatment. Under flow and a VEGF gradient (bottom row), LMB treatment results in reduced sprouting and invasion. Dashed arrow indicates the direction of flow. Control (DMSO) devices (both static and flow) proceeded with extensive sprouting. (Scale bar, 100 μm). (**b**) Quantification of the % of invasion area under static and flow in response to 24 h of continuous leptomycin B (LBM) treatment. Data represent mean ± SEM; *p < 0.05.

Interestingly, our results showed that interstitial flow resulted in increased localization of t- and p-HDAC1 to the cytoplasm (Fig. 3e), as quantified by the area fraction of cytoplasmic t- and p-HDAC1 (Fig. 3f). Inhibition of CRM1 (exportin 1)-dependent transport with leptomycin B (LMB) (20 nM) prevented the increase in cytoplasmic localization (Fig. 3e,f), suggesting that nuclear export of HDAC1 plays a role in the cytoplasmic p-HDAC1 localization. To examine whether nuclear export of HDAC1 is required for angiogenic sprouting, ECs in the microfluidic device were continuously treated with LMB (20 nM) for 24 h in the presence of interstitial flow or under static conditions. Exogenous VEGF was also included in the media. Under flow conditions, inhibition of protein nuclear export significantly reduced invasion into the collagen matrix (Fig. 4a,b), while under static conditions it had no significant effect (Fig. 4a,b). Together these results suggest that CRM1-mediated nuclear export of HDAC1 is part of the mechanism that modulates interstitial flow-induced angiogenic sprouting.

### HDAC1 regulates MMP14 expression in flow-induced angiogenic sprouting

**Figure 5:**
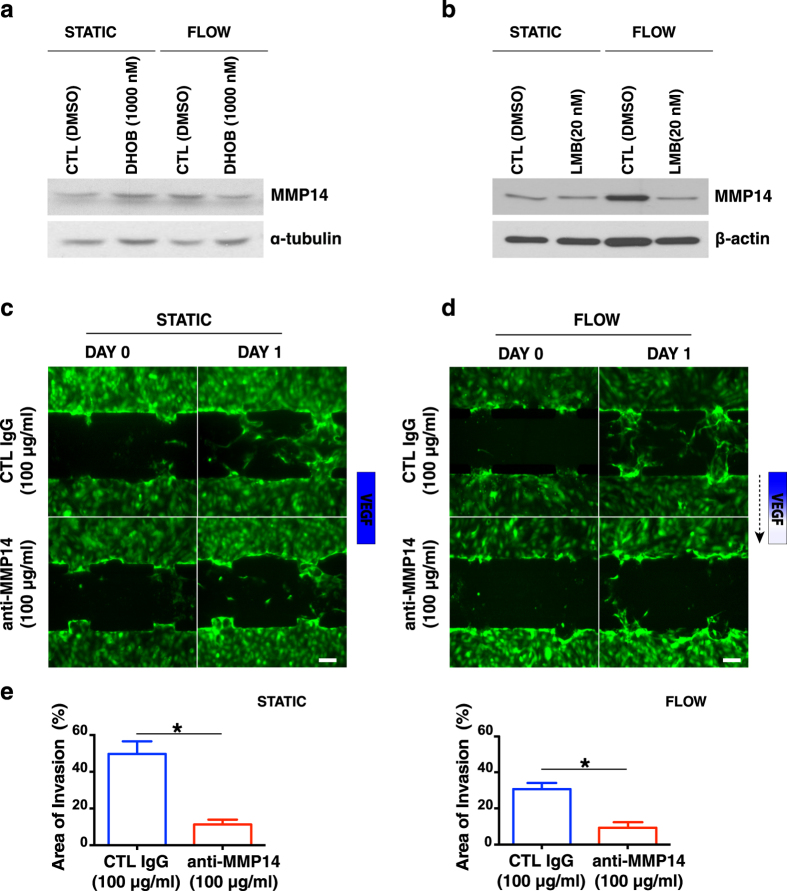
MMP14 expression is regulated by HDAC1. (**a**) Representative western blot showing that HDAC1 blockade with DHOB (1000 nM) significantly reduced MMP14 expression after 1 h of treatment under flow but not under static conditions. The blot was cropped and the full-length blot is presented in Supplementary Fig. S1. (**b**) Representative western blot showing that inhibition of HDAC1 nuclear export with LMB (20 nM) significantly reduced MMP14 expression after 1 h of treatment under flow but not under static conditions. (**c**) MMP14 selective blockade (100 μg/ml anti-MMP14) resulted in significant reduction of invasion under static (no VEGF gradient) and (**d**) flow (dashed arrow indicates the direction of flow) and VEGF gradient (bottom row) conditions. Control (CTL-100 μg/ml IgG) devices (both static and flow) proceeded with extensive sprouting. (Scale bar, 100 μm). (**e**) Quantification of the % area of invasion further supports the microscopic observations. Data represent mean ± SEM, *p < 0.05.

We next examined potential downstream effectors involved in sprouting angiogenesis. It is known that MMPs are intimately involved in the angiogenic process, and MMP14 is expressed by endothelial tip cells^38^,^39^. We found that MMP14 expression increased when HUVECs were subjected to intersitital flow for 1 h (Fig. 5a). We thus assessed whether MMP14 expression in response to flow is dependent on HDAC1. We found that inhibition of HDAC1 with DHOB reduced the increase in MMP14 expression induced by flow (Fig. 5a). Furthermore, inhibition of protein nuclear export with LMB also downregulated MMP14 protein levels after flow (Fig. 5b), suggesting the increase in MMP14 expression after flow may be co-regulated by HDAC1 nuclear export. We then performed our functional assay in the microfluidic channels, further assessing the role of MMP14 in flow-induced angiogenic sprouting. Selective targeting of MMP14 significantly inhibited EC sprouting and invasion under both static (Fig. 5c,e) and flow conditions (Fig. 5d,e).

## Discussion

**Figure 6:**
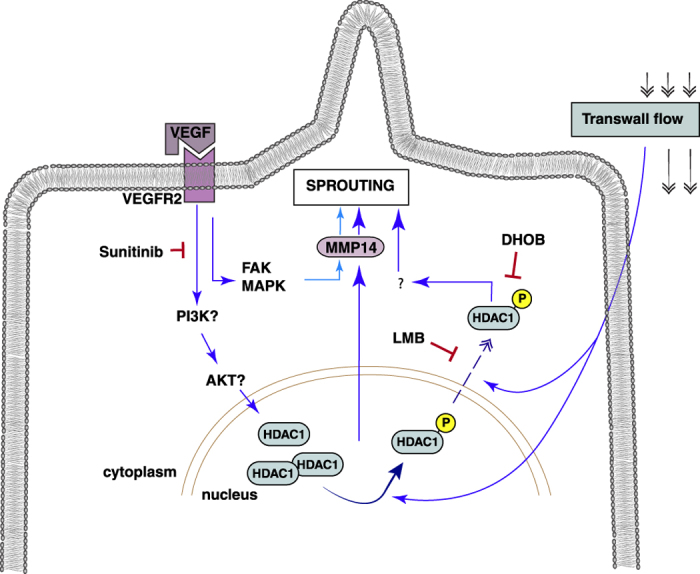
Potential role of HDAC1 in flow-induced angiogenesis. Interstitial (Transwall) flow and VEGF result in increased HDAC1 phosphorylation in the nucleus as well as HDAC1 nuclear export. Nuclear export (dashed blue line) can be reduced with LMB treatment while HDAC1 activity, but not phosphorylation, can be reduced via DHOB treatment. HDAC1 increases MMP14 expression, potentially through its role as a transcriptional regulator. VEGFR2 phosphorylation is integral for both static and flow-induced angiogenic sprouting as revealed by the blocking experiments with sunitinib. HDAC1 appears to complement and amplify the VEGF pathway when flow signals are present.

Extravasating or intravasating plasma creates forces that affect endothelial cells in the vessel wall, but it is not clear how the mechanobiological signals are propagated. Our results show that HDAC1 is a key component of this mechano-signaling pathway. In functional assays, inhibition of HDAC1 suppressed EC morphogenesis under flow but not under static conditions, and interstitial flow increased the phosphorylation of HDAC1 at Ser421 as well as its activity. The observed increase in activity after phosphorylation is consistent with other reports^35^. Although fluid forces activate morphogenesis, this mechanism is also dependent on VEGFR2 activation, as blocking this pathway completely inhibited morphogenesis in our system, regardless of the presence of flow. Furthermore, flow did not increase HDAC1 phosphorylation in the absence of VEGFR2 activation. These results suggest that HDAC1 acts downstream of VEGF in its regulation of angiogenic invasion by activating additional effectors or amplifying existing pathways (Fig. 6).

Previous studies have shown that other members of the HDAC family are involved in the control of EC gene expression and function in response to mechanical stimuli^18^. For example, laminar shear stress at 24 dynes/cm^2^ induces phosphorylation-dependent nuclear export of HDAC5 and its dissociation from MEF2, leading to induction of KLF2 and endothelial nitric oxide (NO) synthase (eNOS)^27^. Oscillatory shear stress can induce HDAC3 phosphorylation in ECs to modulate their survival and integrity^29^. Oscillatory shear stress can also induce sustained expression and accumulation of class I HDACs, while pulsatile shear stress induces phosphorylation-dependent nuclear export of HDACs 5 and 7 in ECs^25^. It remains to be determined whether the various members of the HDAC family perform different functions during EC morphogenesis, or how they coordinate the responses to mechanical stimuli.

Here, we found that HDAC1 export to the cytoplasm is a prerequisite for flow-induced EC morphogenesis. In response to transwall flow, there were increases in both t-HDAC1 and p-HDAC1 in the cytoplasm that could be blocked by LMB treatment. The resulting decrease in cytoplasmic HDAC1 was associated with less morphogenic invasion by the ECs in the functional assay. Although class I HDACs such as HDAC1 are thought to be primarily nuclear enzymes, recent reports have demonstrated that HDAC1 can indeed have cytoplasmic functions. For example, Kim *et al*. reported that HDAC1 is exported from the nucleus via interaction with CRM-1 in damaged axons in brains of multiple sclerosis patients^36^. This is consistent with our findings, as CRM-1 is the cellular target of LMB. HDAC1 has also been reported to interact with the actin binding protein profilin-2 in the cytoplasm, which facilitates its nuclear translocation and therefore its transcriptional repression activity^37^. It is not yet known what cytoplasmic function HDAC1 performs in response to flow, but given the role of the cytoskeleton in mechanotransduction, it may be related to the ability of HDAC1 to interact with microtubules^36^, actin^40^ and actin-binding protein^37^.

Our studies also indicated that MMP14 acts downstream of HDAC1 in flow-induced sprouting. Although MMP14 activity is involved in angiogenic sprouting in both static and flow conditions, we showed that flow can upregulate MMP14 protein expression, and that inhibition of HDAC1 with DHOB attenuates this upregulation (Fig. 5a). This is consistent with previous studies showing that class I HDACs can regulate MMP gene expression^41^,^42^,^43^,^44^,^45^,^46^, with one report specifically indicating that MMP14 gene expression is down-regulated after treatment with a class I HDAC inhibitor^41^. Interestingly, we found that inhibition of HDAC1 export with LMB reduces the increase in MMP14 protein expression in response to flow. This suggests that HDAC1 regulation of MMP14 may not be limited to its nuclear activity, but may also be related to its cytoplasmic accumulation. Given the short time frame of the experiments performed (lysates were collected 1 h after DHOB or LMB inhibition in the presence or absence of flow), it is possible that the regulation of MMP14 expression in response to flow lies beyond gene transcription and translation, further supporting the potential involvement of the cytoplasmic pool of t-HDAC1 and p-HDAC1. It will be interesting for future studies to elucidate if and how HDAC1 regulates MMP14 protein expression in response to flow to effect EC morphogenesis.

However, our results do not preclude the possibility that HDAC1 and MMP14 are simply co-regulated by other molecular mechanisms in response to flow. For example, HDAC1 is known to be regulated by associated proteins including retinoblastoma tumor suppressor (Rb), metastasis-associated protein 2 (MTA2), and nuclear hormone receptors like the retinoic acid receptor^47^, and its enzymatic activity is dependent on complex formation with Sin3, NuRD and CoREST. Casein kinase 2 (CK2) is the only known kinase to phosphorylate HDAC1 at Ser421^22^,^35^,^40^, and although CK2 is reported to be a constitutively active kinase, it is possible that CK2 may also respond to flow, given its role in VEGF signaling^48^,^49^,^50^. It is currently unknown whether any of these HDAC1 associated proteins is involved in flow-induced angiogenesis. As elevated interstitial flow and abnormal angiogenesis are extensive in pathologies such as cancer, understanding how ECs sense and respond to flow via HDAC1 will not only advance our knowledge of the role of mechanotranduction and chromatin remodeling in angiogenesis, but also open up the possibilities of designing novel anti-angiogenic drugs and regimens to advance cancer therapies. As HDAC1 activity and phosphorylation also regulate many other cellular processes such as cell proliferation, differentiation and migration, the understanding of mechanical regulation of HDAC1 activity will also provide mechanistic insights into a range of cell functions beyond pathological angiogenesis.

## Methods

### HUVEC preparation

Human umbilical vein endothelial cells (HUVECs) were acquired from the Center for Excellence in Vascular Biology, Brigham & Women’s Hospital, Harvard Medical School, Boston, MA and maintained in EGM medium (2% FBS, brain bovine extract, heparin, hEGF, and hydrocortisone) (Lonza). HUVECs (passage number 1–5) were used non-labelled or labelled with the Cell Tracker Green CMFDA probe (5 μM) (Invitrogen, Grand Island, NY) as per experimental requirements.

### Microfluidic device fabrication and HUVEC seeding

The microfluidic device for reproducing sprouting angiogenesis under conditions of controlled fluid flow and VEGF gradient was fabricated as previously described^17^. Briefly, each device consisted of two layers of 12:1 base to curing agent poly(dimethylsiloxane) (PDMS, Sylgard 184, Dow Corning). The top layer was constructed using soft lithography to form the negative relief features ~50 μm in height. The top layer with the features was irreversibly sealed against a planar PDMS layer (~2 μm thick) via treatment with plasma oxygen (Harrick, Ithaca, NY) for 60 sec, heated to 60 °C for 30 min, and then sterilized by exposure to UV light for ~30 min.

At least 2 h after plasma oxygen treatment, a mixture of collagen gel (3 mg ml^−1^, rat tail-type I, BD Biosciences, Franklin Lakes, NJ) and fibronectin (10 μg ml^−1^, BD Biosciences) was introduced into one port of the central channel and aspirated through the channel using vacuum pressure at 4 °C to retard collagen polymerization. The collagen gel solution was then allowed to polymerize at 37 °C and hydrated conditions for 48–72 h. Subsequently, the channel regions of the device not containing collagen gel were coated with a fibronectin (FN) solution (10 µg ml^−1^ for 3 h). A concentrated solution (~10^7^ cells/ml) of Cell Tracker Green CMFDA labeled-HUVECs was introduced into the entire length of both the upper and lower channels. The HUVECs attach to the channel walls and then spread and migrate to cover all surfaces of the channels, including the collagen exposed along the array of vertical apertures (Fig. 1a); it is at these interfaces that sprouting can occur. The HUVECs were grown to confluence (24 h after seeding) with passive pumping^51^ of EGM medium. The system recreates two quiescent blood vessels separated by 300 μm with multiple regions for potential endothelial sprouting.

### Control of fluid flow in the microfluidic device

Fluid flow was controlled with a programmable syringe pump (Harvard Apparatus). The flow medium was the same as the growth medium (EGM). Before initiation of flow-based experiments, clear polypropylene barbed elbow fittings (1/16 inch, Cole-Parmer) connected to silicone tubing (Saint-Gobain) were inserted into the 1.5-mm diameter inlet/outlet ports of the HUVEC channels. The opposite inlet/outlet ports of the HUVEC channels were connected to luer adapters (Cole-Parmer) which served as fluid reservoirs. To subject endothelial cells solely to interstitial flow (transverse convection), we pulled media through the collagen matrix from the reservoirs connected to the other endothelial-lined channel, while exposing both channels to negligible levels of tangential shear. A pressure gradient across the collagen gel is also generated. In this configuration, fluid extravasates from one HUVEC channel, convects through the collagen and intravasates into the other channel. The flow rate was 7.3 μl/h corresponding to a flow velocity of 60 μm/sec. Devices without imposed flow served as controls (“static”). In this case, the media was changed every day.

### Macroscale interstitial flow device

To expose a larger number of cells to trans-wall flow for protein or RNA analyses, we constructed the device shown in Fig. 1d. 500 μl of collagen (3 mg ml^−1^) and fibronectin (10 □g ml^−1^) were loaded into a 6-well cell culture insert with 0.4 μm pore-size membrane (Corning, USA). The gel was allowed to polymerize at 37 °C for 2 h. 2 ml of HUVECs (non-labeled) at a concentration of 10^6^/ml in EGM media were then added slowly on top of the collagen gel to prevent gel disruption. Cells were allowed to spread and form a confluent monolayer for 24 h prior to flow initiation.

### Control of fluid flow in the macroscale interstitial flow device

Cells in the macroscale device were subjected to flow for 1 h using a programmable syringe pump (Harvard Apparatus) (Fig. 1d). The flow rate for both configurations was 0.7 ml/h corresponding to a bulk flow velocity of 0.5 μm/sec. The flow medium was the same as the growth medium (*i.e*. EGM). Exogenous VEGF (50 ng/ml) was added to the media as per experimental requirements. Gels not exposed to flow served as control (“static”).

### Pharmacological inhibition of endothelial morphogenesis

To investigate the role of HDAC-1 in endothelial morphogenesis, cells were treated with the HDAC-1-selective inhibitor 4-(dimethylamino)-N-[6-(hydroxyamino)-6-oxohexyl]-benzamide (Santa Cruz Biotechnology, Inc. USA), hereafter denoted as *DHOB*. DHOB was continuously introduced into the HUVEC-lined channels of the microfluidic device by flow (60 μm/sec) for 24 h at concentrations of 30, 100 or 1000 nM. In control devices without flow (“static”), DHOB was added to the luer reservoirs connected to the four ports of the device and allowed to passively diffuse into the HUVEC channels.

Nuclear export of HDAC1 was blocked with leptomycin B (LMB) (20 nM) (Santa Cruz Biotechnology, Inc. USA), a pharmacological inhibitor of CRM1 (exportin 1) – dependent transport. VEGFR2-Tyr951 inhibition was performed with the tyrosine receptor kinase inhibitor Sunitinib (LC Laboratories, USA) at 1000 nM. MMP14 activity was inhibited with a specific anti-MMP14 antibody (Dyax, USA) at a concentration of 100 μg/ml. The experimental protocols for Sunitinib and anti-MMP antibody were as described for DHOB above. All treatments described here were performed in EGM supplemented with 50 ng/ml of exogenous VEGF.

### Phosphorylation profiling

Phosphorylation profiling was performed using the Phospho Explorer Antibody Array (ELISA-based) from Full Moon Biosystems (CA, USA). This array consists of 1318 antibodies from over 30 signaling pathways relevant to our study; it assays for serine, threonine and tyrosine phosphorylation. 2 mg of protein collected from cells exposed to static or flow conditions in the transwell device were sent to Full Moon Biosystems for automated analysis.

### siRNA HDAC1 transfection

Accell SMARTpool siRNA against HDAC1 (E-003493-00) was purchased from Dharmacon (CO, USA). HUVECs were seeded in 25 cm^2^ flasks and then transfected with siRNA HDAC1 or with non-targeting siRNA SMARTpool (Dharmacon D-001950-01) according to the manufacturer’s instructions. 24 h post-transfection cells were seeded on one channel of the device and allowed to spread and form a confluent monolayer for 24 h prior to flow initiation.

### Western blot analysis

HUVEC monolayers (static and flow) in the macroscale flow device were harvested in lysis buffer and clarified by centrifugation. The protein concentrations in the lysates were determined using the BioRad protein assay. 40 μg of whole lysate was applied to SDS-PAGE and transferred to a Hybond PVDF membrane (GE Health), which was then incubated with antibodies to HDAC1 (1:1000, Cell Signaling Technology), HDAC1-pSer421 (1:500, Abcam), VEGFR2 (1:1000, Cell Signaling Technology), VEGFR2-Tyr951 (1:1000, Cell Signaling Technology), MMP14 (1: 2000, AbCAM), and Cleaved-caspase 3 (1:1000, Cell Signaling). β-actin, α-tubulin or GAPDH were used as loading controls. The bound primary antibodies were detected using a horseradish peroxidase (HRP)-conjugated secondary antibody and the ECL detection system (GE Health). Band density was quantified using the Image J software (NIH, Bethesda, MA).

### HDAC1 activity assay

HDAC1 activity assay was performed with the Immunoprecipitation and activity assay from Biovision (CA, USA) as per manufacturer’s instructions. Briefly, HDAC1 was immunoprecipitated from 100 μg of HUVEC cell lysate with rabbit anti-HDAC1 antibody. The beads with antibody-HDAC1 complex were washed three times with PBS, centrifuged for 10 s at 14,000 g before assaying for HDAC1 activity.

### Immunofluorescence staining

EC monolayers exposed for 1 h to interstitial flow in the macroscale device were initially washed with Phosphate Buffered Saline (PBS) to remove any cell media, fixed with 4% formaldehyde for 20 min, washed three times with PBS and subsequently permeabilized with 0.5% Triton in PBS for 10 min at 4 °C. Co-cultures were then rinsed three time with PBS-Glycine followed by serum blockade (5% donkey serum/0.1% Triton in PBS for 60 min). Monolayers were labeled with anti-HDAC1 (1:200 Rockland Immunochemicals Inc.) and anti-pSer421-HDAC1 (1:200, Abcam) antibodies overnight at 4 °C. Appropriate fluorescently labelled secondary antibody was applied for 60 min and washed three times with saline. Cell nuclei were stained with DAPI nuclear stain (Invitrogen, 1:200) and washed again three times with saline prior to confocal microscopy. ECs in the microfluidic device were stained for Notch 1 (1:100, Abcam), Dll4 (1:50, R&D Systems) and Alexa Fluor 488- Phalloidin (2 U/ml) according to the aforementioned protocol.

### Image acquisition

Phase contrast and corresponding fluorescence images were acquired with an Olympus IX81 microscope (20× air lens with 2 μm slice thickness) equipped with an automated stage and the OpenLab software. The area of cell invasion into the collagen gel was quantified using the ImageJ analysis software. Briefly, the area of invasion from each aperture of the microfluidic device was measured at day 1 and then normalized relative to the invasion at day 0, with the exception of the leptomycin B experiments where non-normalized data are illustrated. Projections of confocal images were produced using ImageJ. For the analysis of the t- and p-HDAC1 cytoplasmic staining, we identified the nuclear area (defined as ‘DAPI-positive’ area) and created a binary image where the value 0 was assigned to the nuclear area. Subsequently, we multiplied the DAPI binary image with the t- and p-HDAC1 binary images, eliminating the nuclear region from our analysis. The area fraction of t- and p-HDAC1 outside the nucleus per field of view was quantified and normalized to the total number of cells in each field of view.

### Statistical analysis

The data are shown as mean ± SEM. Analysis of means was performed with a one-tailed t-test or one-way analysis of variance (ANOVA) (GraphPad Prism software, San Diego, CA, USA). Differences were considered significant at P values less than 0.05.

## Additional Information

**How to cite this article**: Bazou, D. *et al*. Flow-induced HDAC1 phosphorylation and nuclear export in angiogenic sprouting. *Sci. Rep*. **6**, 34046; doi: 10.1038/srep34046 (2016).

## Supplementary Material

Supplementary Information

## References

[b1] Carmeliet P. & Jain R. K. Angiogenesis in cancer and other diseases. Nature 407, 249–257, doi: 10.1038/35025220 (2000). 11001068

[b2] Risau W. Mechanisms of angiogenesis. Nature 386, 671–674, doi: 10.1038/386671a0 (1997). 9109485

[b3] Carmeliet P., De Smet F., Loges S. & Mazzone M. Branching morphogenesis and antiangiogenesis candidates: tip cells lead the way. Nat Rev Clin Oncol 6, 315–326, doi: 10.1038/nrclinonc.2009.64 (2009). 19483738

[b4] Gerhardt H. . VEGF guides angiogenic sprouting utilizing endothelial tip cell filopodia. The Journal of cell biology 161, 1163–1177, doi: 10.1083/jcb.200302047 (2003). 12810700 PMC2172999

[b5] Davies P. F. Flow-mediated endothelial mechanotransduction. Physiol Rev 75, 519–560 (1995). 7624393 10.1152/physrev.1995.75.3.519 PMC3053532

[b6] Ingber D. E. Mechanical signaling and the cellular response to extracellular matrix in angiogenesis and cardiovascular physiology. Circulation research 91, 877–887 (2002). 12433832 10.1161/01.res.0000039537.73816.e5

[b7] Patan S., Munn L. L. & Jain R. K. Intussusceptive microvascular growth in a human colon adenocarcinoma xenograft: a novel mechanism of tumor angiogenesis. Microvascular research 51, 260–272, doi: 10.1006/mvre.1996.0025 (1996). 8778579

[b8] Olsson A. K., Dimberg A., Kreuger J. & Claesson-Welsh L. VEGF receptor signalling - in control of vascular function. Nat Rev Mol Cell Biol 7, 359–371, doi: 10.1038/nrm1911 (2006). 16633338

[b9] Mammoto A., Mammoto T. & Ingber D. E. Mechanosensitive mechanisms in transcriptional regulation. Journal of cell science 125, 3061–3073, doi: 10.1242/jcs.093005 (2012). 22797927 PMC3434847

[b10] Sun X. . Activation of integrin alpha5 mediated by flow requires its translocation to membrane lipid rafts in vascular endothelial cells. Proceedings of the National Academy of Sciences of the United States of America 113, 769–774, doi: 10.1073/pnas.1524523113 (2016). 26733684 PMC4725528

[b11] Zhou J., Li Y. S. & Chien S. Shear stress-initiated signaling and its regulation of endothelial function. Arteriosclerosis, thrombosis, and vascular biology 34, 2191–2198, doi: 10.1161/ATVBAHA.114.303422 (2014). PMC4169328 24876354

[b12] Shyy J. Y. & Chien S. Role of integrins in endothelial mechanosensing of shear stress. Circulation research 91, 769–775 (2002). 12411390 10.1161/01.res.0000038487.19924.18

[b13] Hernandez Vera R. . Interstitial fluid flow intensity modulates endothelial sprouting in restricted Src-activated cell clusters during capillary morphogenesis. Tissue Eng Part A 15, 175–185, doi: 10.1089/ten.tea.2007.0314 (2009). 18636940 PMC2809657

[b14] Tada S. & Tarbell J. M. Interstitial flow through the internal elastic lamina affects shear stress on arterial smooth muscle cells. American journal of physiology. Heart and circulatory physiology 278, H1589–1597 (2000). 10775138 10.1152/ajpheart.2000.278.5.H1589

[b15] Song J. W., Bazou D. & Munn L. L. Anastomosis of endothelial sprouts forms new vessels in a tissue analogue of angiogenesis. Integrative biology: quantitative biosciences from nano to macro 4, 857–862, doi: 10.1039/c2ib20061a (2012). 22673771 PMC3759296

[b16] Song J. W., Daubriac J., Tse J. M., Bazou D. & Munn L. L. RhoA mediates flow-induced endothelial sprouting in a 3-D tissue analogue of angiogenesis. Lab on a chip 12, 5000–5006, doi: 10.1039/c2lc40389g (2012). 23073300 PMC3490212

[b17] Song J. W. & Munn L. L. Fluid forces control endothelial sprouting. Proceedings of the National Academy of Sciences of the United States of America 108, 15342–15347, doi: 10.1073/pnas.1105316108 (2011). 21876168 PMC3174629

[b18] Zhou B., Margariti A., Zeng L. & Xu Q. Role of histone deacetylases in vascular cell homeostasis and arteriosclerosis. Cardiovasc Res 90, 413–420, doi: 10.1093/cvr/cvr003 (2011). 21233251

[b19] Pons D. . Epigenetic histone acetylation modifiers in vascular remodelling: new targets for therapy in cardiovascular disease. Eur Heart J 30, 266–277, doi: 10.1093/eurheartj/ehn603 (2009). 19147603

[b20] Delcuve G. P., Khan D. H. & Davie J. R. Roles of histone deacetylases in epigenetic regulation: emerging paradigms from studies with inhibitors. Clin Epigenetics 4, 5, doi: 10.1186/1868-7083-4-5 (2012). 22414492 PMC3320549

[b21] Kelly R. D. & Cowley S. M. The physiological roles of histone deacetylase (HDAC) 1 and 2: complex co-stars with multiple leading parts. Biochem Soc Trans 41, 741–749, doi: 10.1042/BST20130010 (2013). 23697933

[b22] Khan D. H. . Protein kinase CK2 regulates the dimerization of histone deacetylase 1 (HDAC1) and HDAC2 during mitosis. The Journal of biological chemistry 288, 16518–16528, doi: 10.1074/jbc.M112.440446 (2013). 23612983 PMC3675587

[b23] Iordache F., Buzila C., Constantinescu A., Andrei E. & Maniu H. Histone deacetylase (HDAC) inhibitors down-regulate endothelial lineage commitment of umbilical cord blood derived endothelial progenitor cells. Int J Mol Sci 13, 15074–15085, doi: 10.3390/ijms131115074 (2012). 23203112 PMC3509628

[b24] Rossig L. . Histone deacetylase activity is essential for the expression of HoxA9 and for endothelial commitment of progenitor cells. J Exp Med 201, 1825–1835, doi: 10.1084/jem.20042097 (2005). 15928198 PMC2213253

[b25] Lee D. Y. . Role of histone deacetylases in transcription factor regulation and cell cycle modulation in endothelial cells in response to disturbed flow. Proceedings of the National Academy of Sciences of the United States of America 109, 1967–1972, doi: 10.1073/pnas.1121214109 (2012). 22308472 PMC3277521

[b26] Wang S. . Control of endothelial cell proliferation and migration by VEGF signaling to histone deacetylase 7. Proceedings of the National Academy of Sciences of the United States of America 105, 7738–7743, doi: 10.1073/pnas.0802857105 (2008). 18509061 PMC2409381

[b27] Wang W. . Fluid shear stress stimulates phosphorylation-dependent nuclear export of HDAC5 and mediates expression of KLF2 and eNOS. Blood 115, 2971–2979, doi: 10.1182/blood-2009-05-224824 (2010). 20042720 PMC2854437

[b28] Yan Z. Q. . Histone deacetylases modulate vascular smooth muscle cell migration induced by cyclic mechanical strain. Journal of biomechanics 42, 945–948, doi: 10.1016/j.jbiomech.2009.01.012 (2009). 19261284

[b29] Zampetaki A. . Histone deacetylase 3 is critical in endothelial survival and atherosclerosis development in response to disturbed flow. Circulation 121, 132–142, doi: 10.1161/CIRCULATIONAHA.109.890491 (2010). 20026773

[b30] Zeng L. . HDAC3 is crucial in shear- and VEGF-induced stem cell differentiation toward endothelial cells. The Journal of cell biology 174, 1059–1069, doi: 10.1083/jcb.200605113 (2006). 16982804 PMC2064396

[b31] Fraccaroli A. . Visualization of endothelial actin cytoskeleton in the mouse retina. PloS one 7, e47488, doi: 10.1371/journal.pone.0047488 (2012). 23115648 PMC3480364

[b32] Phng L. K., Stanchi F. & Gerhardt H. Filopodia are dispensable for endothelial tip cell guidance. Development 140, 4031–4040, doi: 10.1242/dev.097352 (2013). 24046319

[b33] Qazi H., Palomino R., Shi Z. D., Munn L. L. & Tarbell J. M. Cancer cell glycocalyx mediates mechanotransduction and flow-regulated invasion. Integrative biology: quantitative biosciences from nano to macro 5, 1334–1343, doi: 10.1039/c3ib40057c (2013). 24077103 PMC4249644

[b34] Shi Z. D., Ji X. Y., Berardi D. E., Qazi H. & Tarbell J. M. Interstitial flow induces MMP-1 expression and vascular SMC migration in collagen I gels via an ERK1/2-dependent and c-Jun-mediated mechanism. American journal of physiology. Heart and circulatory physiology 298, H127–135, doi: 10.1152/ajpheart.00732.2009 (2010). 19880665 PMC2806139

[b35] Pflum M. K., Tong J. K., Lane W. S. & Schreiber S. L. Histone deacetylase 1 phosphorylation promotes enzymatic activity and complex formation. The Journal of biological chemistry 276, 47733–47741, doi: 10.1074/jbc.M105590200 (2001). 11602581

[b36] Kim J. Y. . HDAC1 nuclear export induced by pathological conditions is essential for the onset of axonal damage. Nature neuroscience 13, 180–189, doi: 10.1038/nn.2471 (2010). 20037577 PMC2829989

[b37] Tang Y. N. . Epigenetic regulation of Smad2 and Smad3 by profilin-2 promotes lung cancer growth and metastasis. Nature communications 6, 8230, doi: 10.1038/ncomms9230 (2015). 26354229

[b38] Koziol A. . The protease MT1-MMP drives a combinatorial proteolytic program in activated endothelial cells. FASEB journal: official publication of the Federation of American Societies for Experimental Biology 26, 4481–4494, doi: 10.1096/fj.12-205906 (2012). 22859368

[b39] Yana I. . Crosstalk between neovessels and mural cells directs the site-specific expression of MT1-MMP to endothelial tip cells. Journal of cell science 120, 1607–1614, doi: 10.1242/jcs.000679 (2007). 17405818

[b40] He S., Khan D. H., Winter S., Seiser C. & Davie J. R. Dynamic distribution of HDAC1 and HDAC2 during mitosis: association with F-actin. Journal of cellular physiology 228, 1525–1535, doi: 10.1002/jcp.24311 (2013). 23280436

[b41] Chen S. . Histone deacetylase (HDAC) activity is critical for embryonic kidney gene expression, growth, and differentiation. The Journal of biological chemistry 286, 32775–32789, doi: 10.1074/jbc.M111.248278 (2011). 21778236 PMC3173185

[b42] Fanjul-Fernandez M., Folgueras A. R., Cabrera S. & Lopez-Otin C. Matrix metalloproteinases: evolution, gene regulation and functional analysis in mouse models. Biochimica et biophysica acta 1803, 3–19, doi: 10.1016/j.bbamcr.2009.07.004 (2010). 19631700

[b43] Jeon H. W. & Lee Y. M. Inhibition of histone deacetylase attenuates hypoxia-induced migration and invasion of cancer cells via the restoration of RECK expression. Molecular cancer therapeutics 9, 1361–1370, doi: 10.1158/1535-7163.MCT-09-0717 (2010). 20442303

[b44] Ma Z., Shah R. C., Chang M. J. & Benveniste E. N. Coordination of cell signaling, chromatin remodeling, histone modifications, and regulator recruitment in human matrix metalloproteinase 9 gene transcription. Molecular and cellular biology 24, 5496–5509, doi: 10.1128/MCB.24.12.5496-5509.2004 (2004). 15169910 PMC419859

[b45] Park S. Y. . Histone deacetylases 1, 6 and 8 are critical for invasion in breast cancer. Oncol Rep 25, 1677–1681, doi: 10.3892/or.2011.1236 (2011). 21455583

[b46] Whetstine J. R. . Regulation of tissue-specific and extracellular matrix-related genes by a class I histone deacetylase. Mol Cell 18, 483–490, doi: 10.1016/j.molcel.2005.04.006 (2005). 15893731

[b47] Cress W. D. & Seto E. Histone deacetylases, transcriptional control, and cancer. Journal of cellular physiology 184, 1–16, doi: 10.1002/(SICI)1097-4652(200007)184:1<1::AID-JCP1>3.0.CO;2-7 (2000). 10825229

[b48] Morooka S. . Identification of a Dual Inhibitor of SRPK1 and CK2 That Attenuates Pathological Angiogenesis of Macular Degeneration in Mice. Molecular pharmacology 88, 316–325, doi: 10.1124/mol.114.097345 (2015). 25993998

[b49] Mottet D., Ruys S. P., Demazy C., Raes M. & Michiels C. Role for casein kinase 2 in the regulation of HIF-1 activity. International journal of cancer 117, 764–774, doi: 10.1002/ijc.21268 (2005). 15957168

[b50] Pollreisz A. . Retinal pigment epithelium cells produce VEGF in response to oxidized phospholipids through mechanisms involving ATF4 and protein kinase CK2. Experimental eye research 116, 177–184, doi: 10.1016/j.exer.2013.08.021 (2013). 24021586

[b51] Walker G. M. & Beebe D. J. A passive pumping method for microfluidic devices. Lab on a chip 2, 131–134, doi: 10.1039/b204381e (2002). 15100822

