# Supplementary material for: Flow-induced HDAC1 phosphorylation and nuclear export in angiogenic sprouting
Source: Sci Rep. 2016 Sep 27;6:34046. doi: 10.1038/srep34046 (PMC5037418; doi:10.1038/srep34046)

Flow-induced HDAC1 phosphorylation and nuclear export in angiogenic sprouting

[#]Despina Bazou[1], [#]Mei Rosa Ng[1], Jonathan W Song[2], Shan Min Chin[1], Nir Maimon[1], and [*]Lance L Munn[1]

[#]equal contribution

Supplementary Information

Supplementary Fig. S1. Uncropped, unprocessed images of western blots.

Fig. 2e

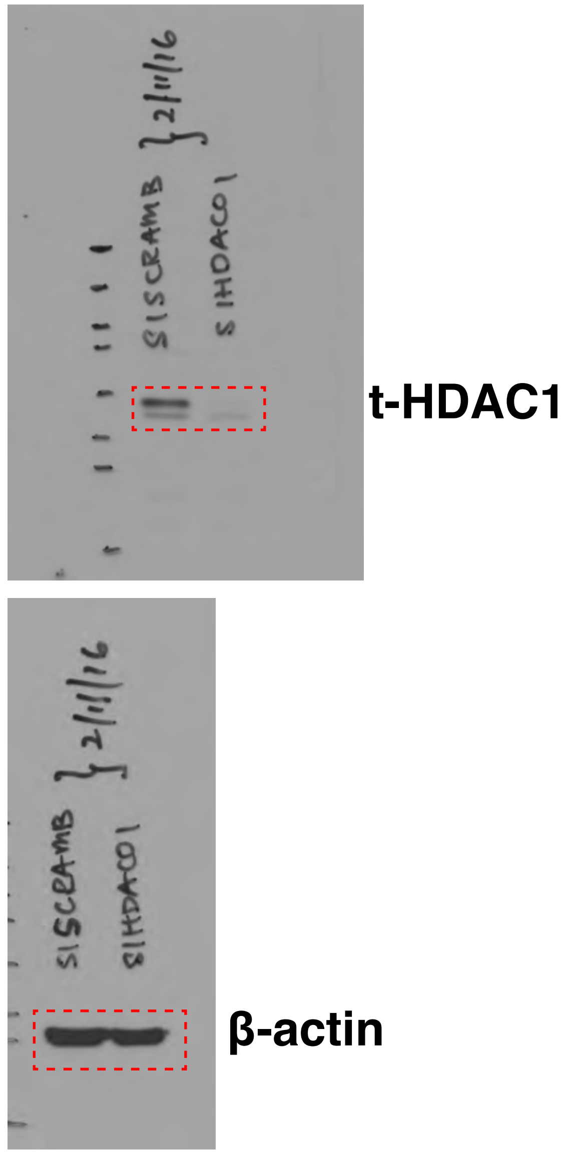

Fig. 3a

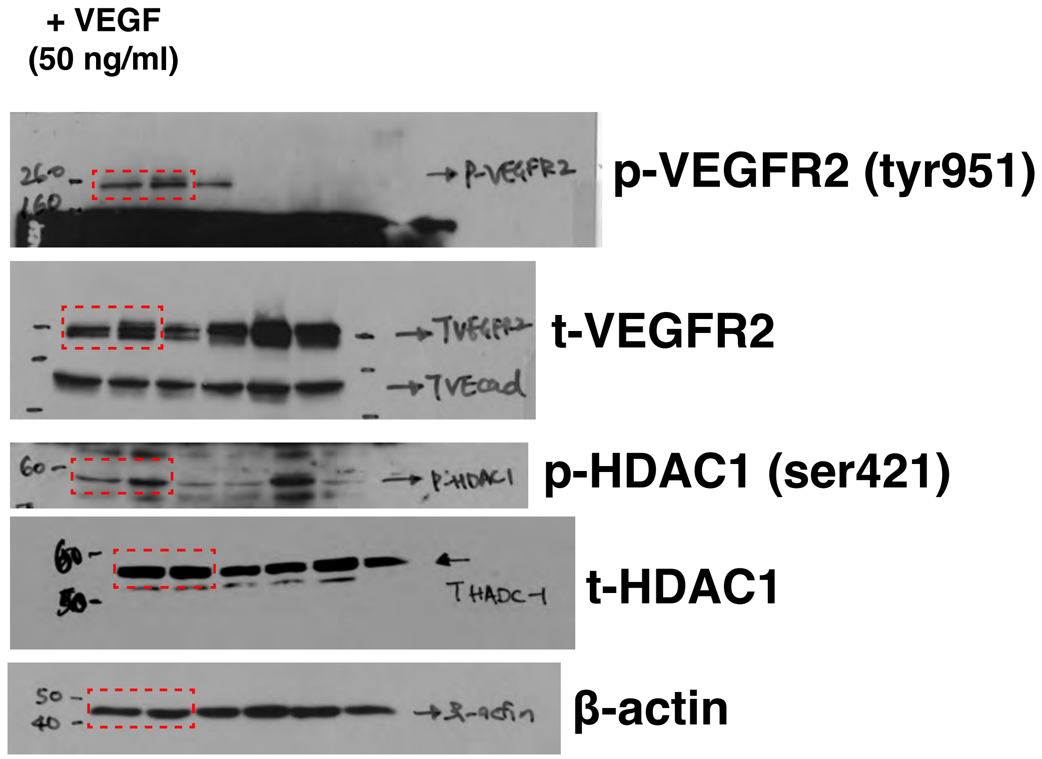

Fig. 3a

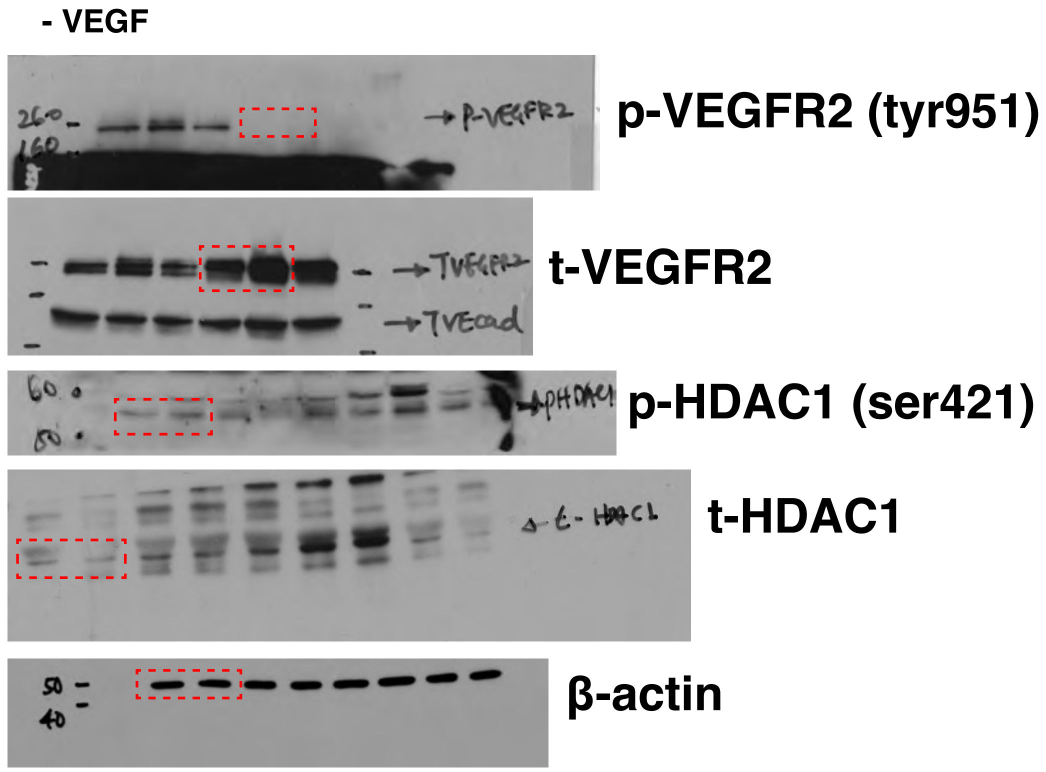

Fig. 5b

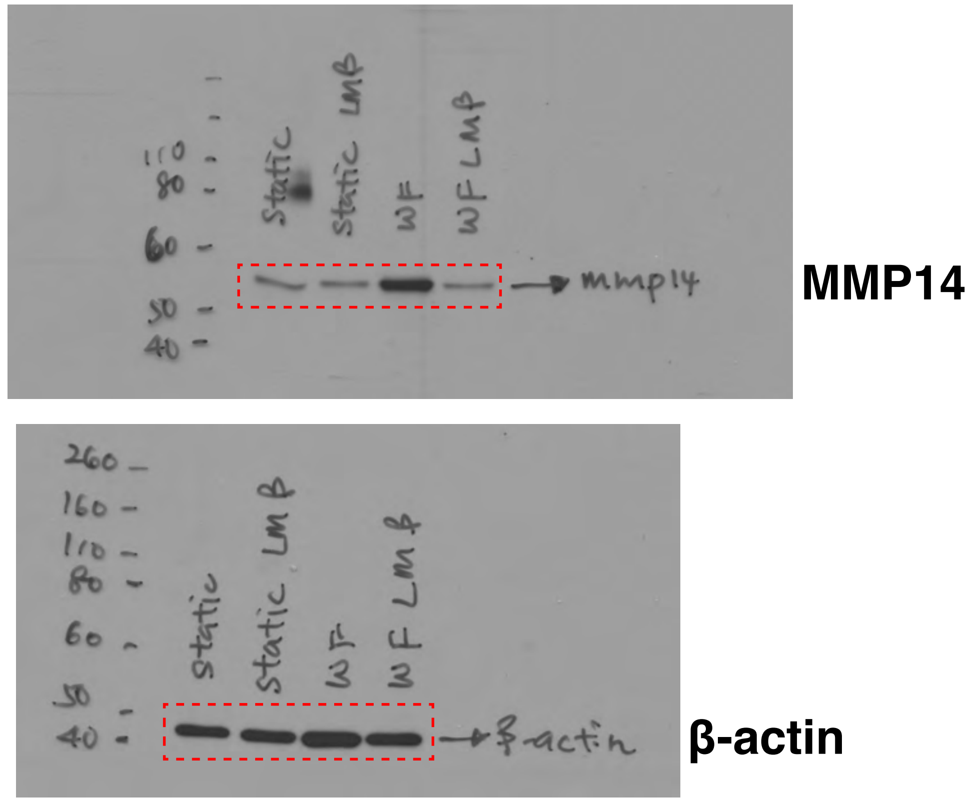

Fig. 3c

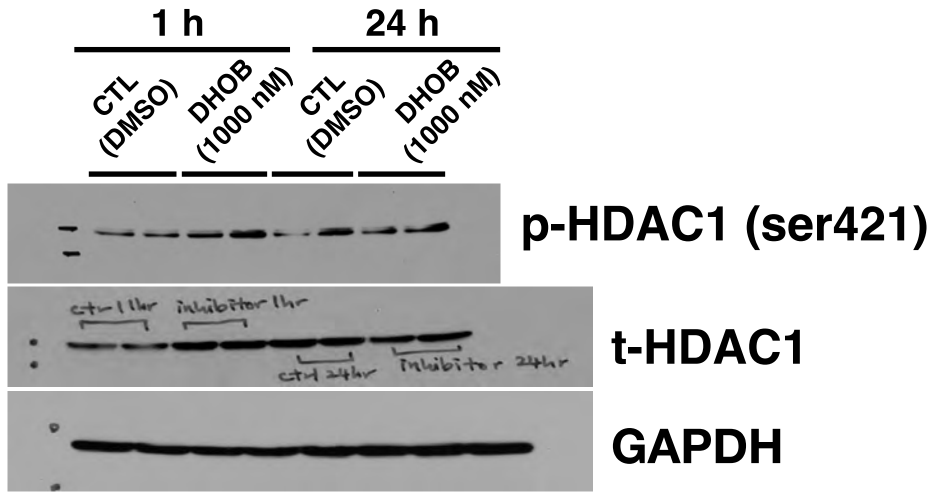

Fig. 5a

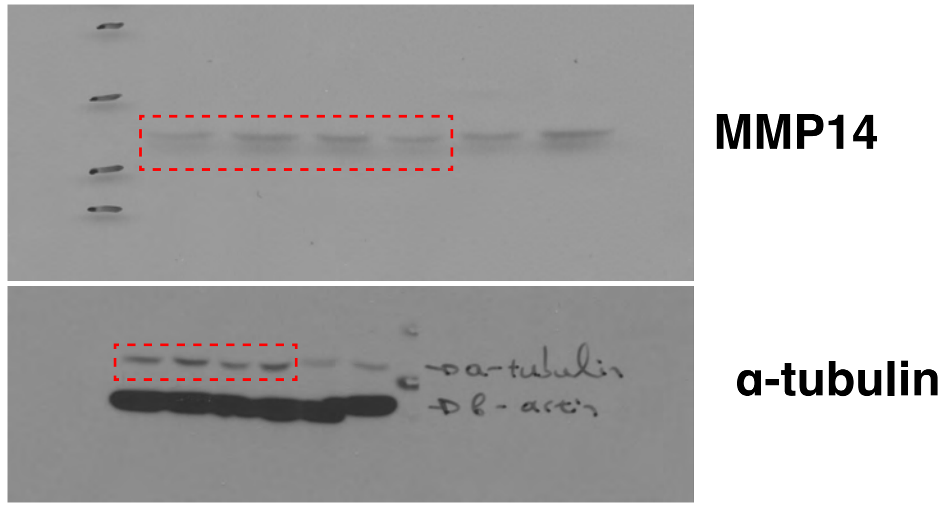

Supplement: Supplementary Information [file srep34046-s1.pdf]
